# Challenging Diagnosis of a Congenital Tracheal Malformation: Considerations From an Intensive Care Perspective

**DOI:** 10.7759/cureus.34404

**Published:** 2023-01-30

**Authors:** Catarina G Morais, Carolina Baptista, Marta Grilo, Amélia Moreira, Augusto Ribeiro

**Affiliations:** 1 Pediatrics, Centro Hospitalar e Universitário de São João, Porto, PRT; 2 Pediatric Intensive Care Unit, Centro Hospitalar e Universitário de São João, Porto, PRT

**Keywords:** tracheal stenosis, congenital abnormalities, pediatric intensive care units, mechanical ventilators, airway resistance

## Abstract

Congenital tracheal stenosis is a rare airway malformation. A high index of suspicion is fundamental. The authors report a case of congenital tracheal stenosis in a 13-month-old male infant, with a challenging diagnosis from the intensive care perspective. At birth, the patient presented an anorectal malformation with a recto-urethral fistula so a colostomy with mucous fistula was performed in the neonatal period. At the age of seven months, he was admitted due to a respiratory infection, treated with steroids and bronchodilators, and discharged after three days without any complications. He underwent complete repair of tetralogy of Fallot when he was 11 months old, which was performed without any reported perioperative complications. However, at the age of 13 months, due to another respiratory infection, he presented more severe symptoms and required admission to the pediatric intensive care unit (PICU) for invasive mechanical ventilation. He was intubated on the first attempt. While monitoring the difference between peak inspiratory and plateau pressures, we observed a sustained elevated difference between pressures suggestive of increased airway resistance, thus raising the possibility of an anatomical obstruction. Laryngotracheoscopy confirmed distal tracheal stenosis (grade II) with four complete tracheal rings. In our case, the absences of perioperative challenges or complications in previous respiratory infections were not suggestive of a tracheal malformation. Furthermore, no difficulties were encountered during intubation due to the distal location of the tracheal stenosis. A careful appreciation of respiratory mechanics on the ventilator at rest and during tracheal aspirations was essential to suspect an anatomical defect.

## Introduction

Congenital tracheal stenosis (CTS) is a rare airway malformation, most frequently secondary to complete tracheal cartilage rings and the absence of the membranous trachea [1,2]. The true incidence remains unknown because of the high mortality prior to a confirmed diagnosis, but it has an estimated incidence of 1 in 64,500 live births [2-5].

Symptoms and signs are largely dependent on the individual patient, their comorbidities, and the location, length, and severity of airway luminal narrowing [2-4,6]. These are responsible for a wide spectrum of clinical presentations that may include a neonatal life-threatening respiratory event, an episode of stridor during childhood, or an unclarified dyspnea in early adolescence [3,4,6]. CTS can be incidentally discovered during a surgical procedure due to unexpectedly difficult airway management, or either upon intubation or in the immediate postoperative period [4]. It can also be revealed in the context of a respiratory tract infection due to additional airway obstruction by secretions and/or bronchospasms [1,4]. Several of those scenarios have been published in recent literature [4,7].

The authors report the case of a CTS in a 13-month-old male infant, with a challenging diagnosis during his hospitalization in the PICU.

## Case presentation

Concerning medical background, the patient’s mother was a 38-year-old woman with a history of miscarriage due to ectopic pregnancy, requiring salpingectomy of the right fallopian tube. The patient was conceived by in vitro fertilization; he was the second twin of a dichorionic diamniotic twin pregnancy. Both twins had fetal growth restriction, but only our patient had echocardiographic findings suggestive of tetralogy of Fallot (TOF), detected at 22 weeks of gestation. Amniocentesis was performed at 22 weeks and four days; results from prenatal array comparative genomic hybridization were unremarkable.

He was born by cesarean delivery at 36 weeks and two days, with an Apgar score of 9/10. He presented an anorectal malformation with recto-urethral fistula, so a colostomy with mucous fistula was performed in the neonatal period. Moreover, postnatal echocardiographic findings were compatible with TOF (peri-membranous ventricular septal defect, ​subaortic, with bidirectional shunting; infundibular and valvular pulmonary stenosis; patent foramen ovale with left-to-right shunting).

At the age of seven months, he was admitted to the pediatrics department due to respiratory distress (increased respiratory rate, retractions, wheezing) in the context of a respiratory infection, without stridor or hypoxemia, with detection of rhinovirus and enterovirus on nasopharyngeal secretions.

He was submitted to complete repair of TOF when he was 11 months old, without any description of perioperative complications (including post-extubation stridor and difficulties in ventilatory weaning).

However, at the age of 13 months, due to another respiratory infection, he presented more severe symptoms; he was admitted to the emergency department with acute respiratory distress, stridor, and oxygen saturation of 80% on room air. High-flow oxygen was administered, and an arterial blood gas was taken, which showed acute hypercapnic respiratory failure - PH: 7.19, PCO2: 9.96 kPa (74.7 mmHg), PO2: 11.73 kPa (88 mmHg), and HCO3: 22.8 mmol/L - requiring admission in our PICU for invasive mechanical ventilation.

**Figure 1:**
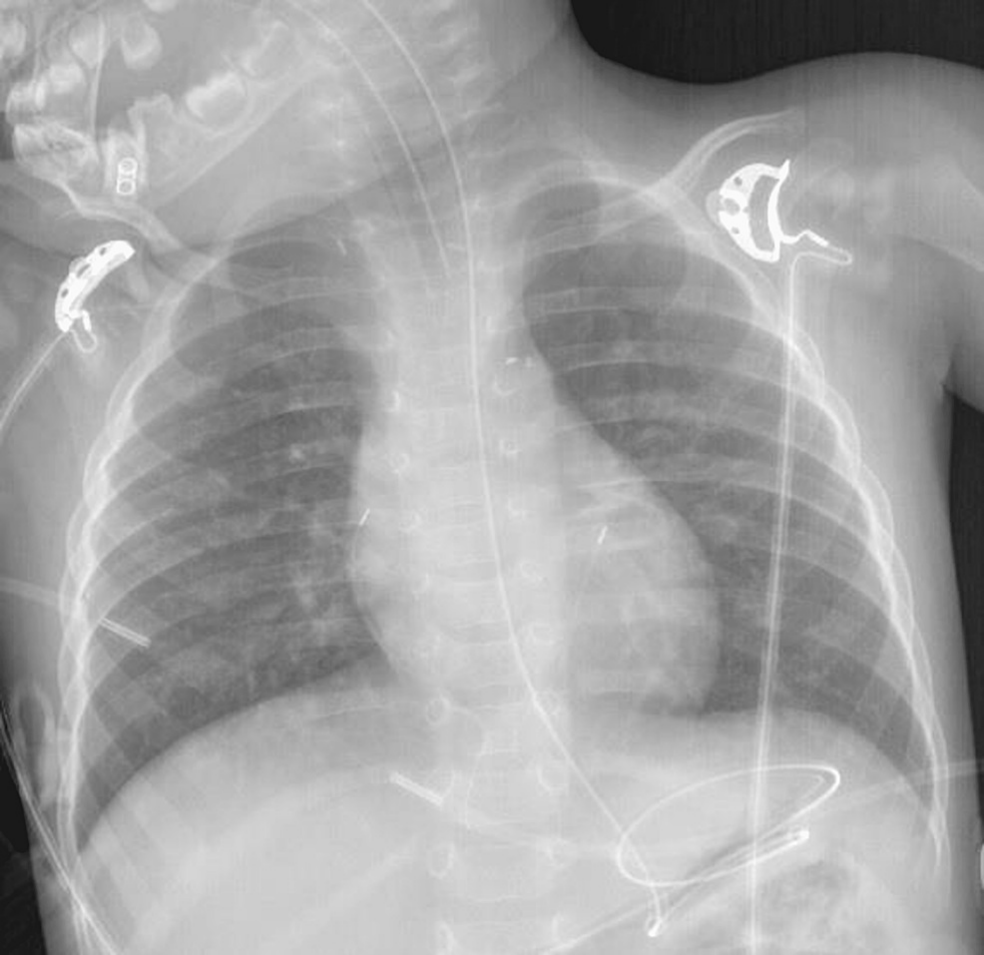
Radiographic evaluation of endotracheal tube position The endotracheal tube was correctly placed. In this chest radiograph, tracheal stenosis is not apparent.

He was intubated on the first attempt with a 4.0 mm oral cuffed endotracheal tube positioned at 13 cm (Figure 1), with normal bilateral chest expansion. 

He was placed on a volume control ventilation mode (initial ventilator settings: tidal volume (Vt) 7 ml/kg, positive end-expiratory pressure (PEEP): 5 cm H2O, rate: 30/min, and FiO2: 60%). The cardiological evaluation did not show signs of acute deterioration. Empirical antibiotic treatment (ceftriaxone), inhaled steroids (budesonide), and bronchodilators (salbutamol, ipratropium bromide) were initiated.

**Figure 2:**
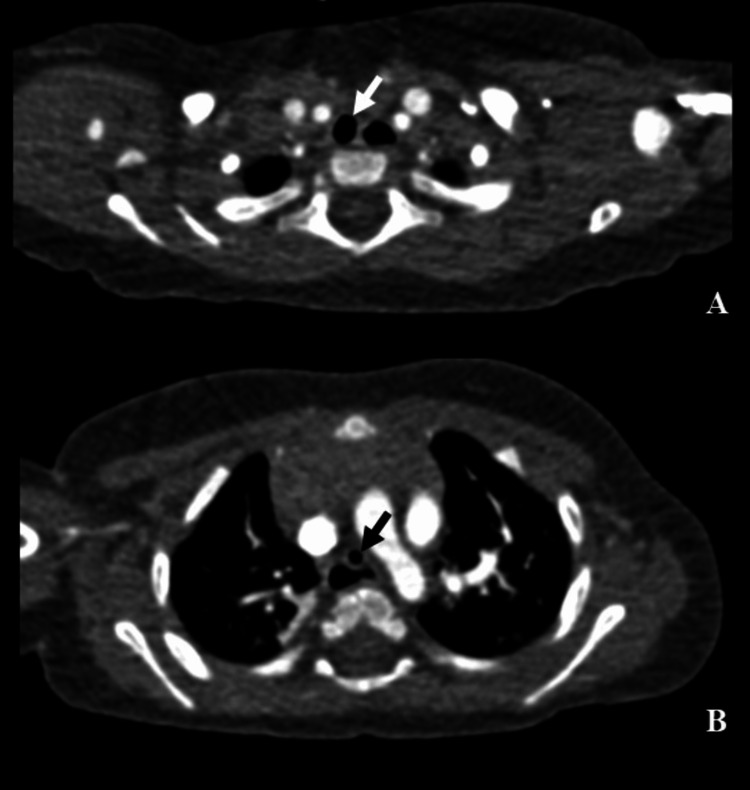
Preoperative computed tomography angiogram of the chest A: Axial view showing a non-stenotic sub-cricoid trachea (arrow). B: Axial view at the thoracic level showing an annular stenotic distal segment of the trachea (arrow).

While monitoring the difference between peak inspiratory (PIP) and plateau (Pplat) pressures, we observed a sustained elevated difference between pressures (∆ PIP-Pplat ~ 10 cm H2O) suggestive of increased airway resistance, with normal inspiratory time and I:E ratio of 1:2. The patient did not exhibit bronchospasms or air trapping. The patient was also evaluated after tracheal aspirations, so airway resistance increase was unlikely due to respiratory secretions. In this context, the possibility of an anatomical obstruction was considered. Therefore, a bronchofibroscopy was performed and revealed an accentuated narrowing of the tracheal lumen, 0.5 cm distal to the tip of the endotracheal tube. This obstruction prevented the passage of the bronchoscope, so the carina was not observed. Carefully reviewing his preoperative imaging for TOF correction, a narrowing of the distal trachea seemed apparent (Figure 2A-2B).

**Figure 3:**
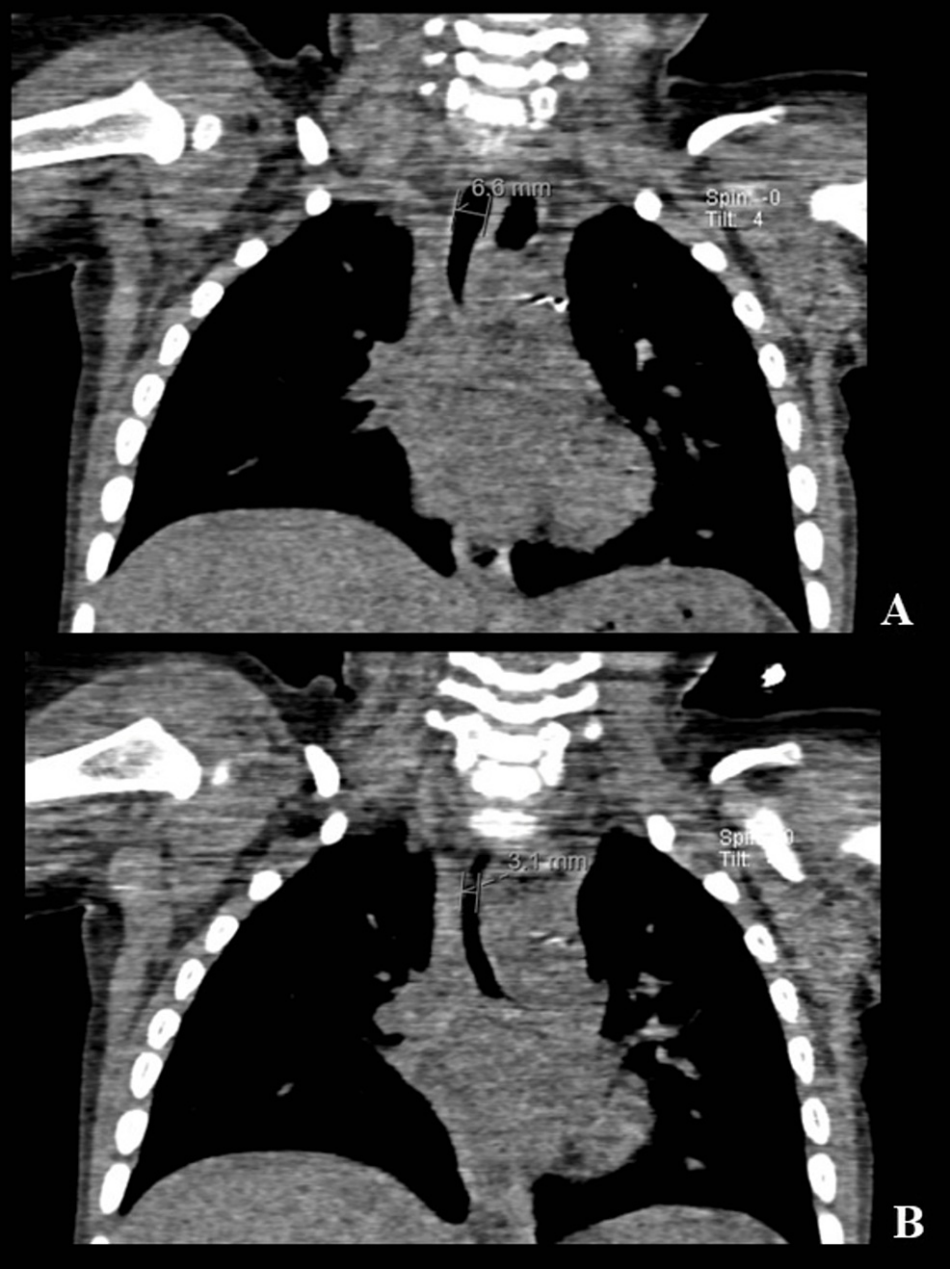
Computed tomography angiogram of the chest A: Coronal view showing a non-stenotic sub-cricoid trachea with a diameter of approximately 6.6 mm. B: Coronal view showing a stenotic distal segment of the trachea with a diameter of approximately 3.1 mm.

Laringotracheoscopy confirmed distal tracheal stenosis (grade II) with four complete tracheal rings. A subsequent contrast chest CT showed an annular stenotic segment of the trachea (Figure 3A-3B) from the level of the third thoracic vertebra to a T-shaped carina, with a reduction in tracheal diameter of more than 50%. Vascular anomalies including pulmonary artery sling were not observed.

He was initiated on therapy with intravenous dexamethasone to limit the development of airway edema and was extubated within 24 hours of the laryngotracheoscopy. He displayed post-extubation stridor that resolved completely with the maintenance of dexamethasone and additional therapy with nebulized adrenaline and heliox. Supplemental oxygen by nasal cannula was necessary for the next 24 hours; he was later discharged to the pediatrics department with a patent natural airway, no signs of respiratory distress or hypoxemia on room air, and normal chest auscultation. Finally, he was referred to an expert center of pediatric tracheal surgery for slide tracheoplasty.

## Discussion

The first classification of CTS by Cantrell in 1964 described three groups according to the length of stenosis: generalized hypoplasia of the trachea, funnel-type stenosis, and segmental stenosis involving short or long segments [6,8]. In the reported case, the sub-cricoid trachea was non-stenotic, but the trachea became progressively narrower toward the carina in a funnel-like shape, so it was formally classified as a CTS with complete rings, grade II according to Cantrell (the most frequent type).

CTS is often associated with other congenital anomalies, mainly cardiac (>50%) but also non-cardiac [4,9]. The complexity of these combined anomalies frequently intensifies operative risk [4]. In our case, before the diagnosis of CTS, the infant had already been submitted to surgical procedures to correct other cardiac (TOF) and non-cardiac (anorectal malformation) anomalies.

Our patient was 13 months old when the diagnosis of CTS was confirmed. As previously mentioned, it is not uncommon to present symptoms for the first time at this age. However, the absence of perioperative challenges or complications in previous respiratory infections was not suggestive of a tracheal malformation. Furthermore, when the patient was admitted to our hospital, no difficulties were encountered during intubation due to the distal location of the tracheal stenosis, meaning a high index of suspicion was needed for this rare diagnosis.

It should also be noted that the presence of cardiac malformations sometimes misleads the diagnosis because it can mimic symptoms of CTS [4]. Even though that was not necessarily a confounding factor in our case, this patient was immediately evaluated by cardiology in the emergency department to exclude an acute decompensation due to their past medical history.

Direct laryngoscopy and flexible bronchoscopy remain the best methods to diagnose and characterize CTS [2,4]. The treatment of CTS, based on functional status, usually requires surgical intervention. Several surgical approaches have been described, however, slide tracheoplasty is now recognized as the gold-standard procedure, irrespective of airway anatomy and length of tracheal stenosis [3,4,9].

## Conclusions

In conclusion, CTS is a rare airway malformation and a high index of suspicion is fundamental. The authors report a challenging diagnosis of CTS in PICU due to the patient's medical background and absence of difficulties during intubation. It was a careful appreciation of respiratory mechanics on the ventilator at rest and during tracheal aspirations that led to a consideration of structural etiology and consequent additional investigation. Slide tracheoplasty is now recognized as the gold-standard surgical procedure and should be performed in specialized centers.
